# Cincinnati Medical Society

**Published:** 1880-04

**Authors:** 


### CINCINNATI MEDICAL SOCIETY.

Dr. A. T. Keyt read the essay of the evening entitled "presphygmic interval"; and exhibited one of his improved sphygmographs, the same that was made for, and will be forwarded to, Professor Marey, of Paris.

The time elasping after the beginning of the systole of the ventricle until the blood begins to escape into the aorta, was termed by Dr. Keyt the presphygmic interval. By means of the instrument, which he exhibited and explained, he had been enabled to arrive at the facts presented in the paper. The instrument consists of two uniform sphygmographs of transmission and a chronograph, combined in one convenient apparatus, and all writing upon the same smoked glass slide. An oblong base closed below by an elastic membrane receives the movements to be inscribed and the latter are transmitted through a flexible tube of convenient length to a small cup closed above with a delicate elastic disk. The movements of the disk correspond with those impressed upon the basal membrane, and they are again conveyed to the writing lever through an upright pin connecting the center of the disk with the lever. Each sphygmograph is also provided with a manometric column which shows the movements to the eye, and measures their range and the degree of pressure required to develop them. The instruments are provided with the requisite stop-cocks and adjusting devices, also a reservoir for the supply of liquid. The chronograph is essentially a watch provided with an inscribed lever which writes correctly fifths of seconds.

The apparatus is convenient and correct. Used singly it traces in a perfect manner the various movements, as the pulsations of the heart and arteries, the respiration, etc., and in its double capacity traces two movements simultaneously, showing their characters and exact time relations.

Among the facts developed in the paper were the following: The interval between the beginning of the heart's impulse against the chest-wall and the beginning of the carotid pulse practically represents the presphygmic. The interval is subject to a limited variation in health at the same pulse-rate, and varies inversely with the pulse-rate. In health, with pulse at 75 per minute, the cardio-carotid interval averages in adults .08 seconds; and approximately the interval may be estimated at one-tenth the duration of the pertaining pulsation.

The mean average velocity of the pulse-wave from the carotid point to the dorsalis pedis is 361 inches per second; from the carotid to the radial, 290 inches per second; from the carotid to the femoral, 269 inches per second; and from the femoral to the dorsalis pedis, 464 inches per second.

On the basis of the carotid-femoral pulse-wave velocity, and seven inches as the arterial length between the aortic root and carotid point, the transit-time of the pulse-wave between these points was computed at .026 second. This value deducted from .08 second, the average cardio-carotid time-difference, gave .54 second as the average presphygmic interval—with pulse at 75.

According to the paper, the cardio-carotid interval varies immensely in disease. It is greatly increased in pure mitral insufficiency, and in aortic obstruction in the form of heavy aortic valves; it is lessened in large aortic insufficiency—the variations in these instances depending upon the variations of the presphygmic interval.

In conclusion, the author remarked: The remarkable range of variation of the cardio-carotid interval in disease, in the face of the comparatively small variation in health, thus demonstrated, opens the way for new and invaluable additions to our diagnostic resources. The few facts presented afford positive and precise indications of the existence and gravity of certain cardiac valvular lesions, and are an earnest of the rich harvest of results that may be expected to follow this line of observation.

Dr. Carson remarked that he had seen two cases in which Dr. Keyt's sphymographic conclusions had been verified by post mortem examinations. One was a case of mitral regurgitation with slight dilatation of the aorta at its base in which there was considerable delay in the pulse as indicated by the tracings. The other was a case of pulsating tumor of the abdomen, which proved to be a malignant growth of the pyloric end of the stomach. The case was not discussed.

